# Ganglioside GM3 in the Tumor Microenvironment: Mechanisms of Signaling Regulation and Strategies for Angiogenesis Inhibition

**DOI:** 10.3390/biom16030464

**Published:** 2026-03-19

**Authors:** Min Zeng, Hongda Zhuang, Siyuan Zhao, Roger Chammas, Yong Chen

**Affiliations:** 1 Institute for Advanced Study, Nanchang University, Nanchang 330031, Chinahongdadazhuang@gmail.com (H.Z.); 2 The MOE Basic Research and Innovation Center for the Targeted Therapeutics of Solid Tumors, School of Pharmacy, Nanchang University, Nanchang 330031, China; 3 Department of Pharmacology, Physiology and Neuroscience, New Jersey Medical School, Rutgers University, Newark, NJ 07103, USA; 4 Center for Translational Research in Oncology, University of São Paulo, São Paulo 01246-000, Brazil

**Keywords:** ganglioside GM3, tumor angiogenesis, VEGF signaling, tumor microenvironment, targeted therapy, HIF-1α

## Abstract

Ganglioside GM3, a fundamental glycosphingolipid on the mammalian cell surface, is a key regulator of transmembrane signaling and cellular recognition. In oncology, GM3 acts as a tumor suppressor by modulating the activity of various receptor tyrosine kinases (RTKs) and their downstream pathways. Recent studies highlight its function in the tumor microenvironment (TME), specifically its ability to impede pathological angiogenesis. This review summarizes the molecular mechanisms by which GM3 interferes with pro-angiogenic signaling, such as the VEGF/VEGFR axis, and discusses how this inhibition can be used for therapy. We explore the clinical potential of GM3-based strategies, including monoclonal antibodies and cancer vaccines, discussing the potential of targeting GM3 to reshape the TME and suppress tumor-associated vascularization.

## 1. Introduction

Tumor angiogenesis is a critical driver of cancer progression and a prerequisite for metastasis. Although anti-angiogenic therapies targeting the Vascular Endothelial Growth Factor (VEGF) pathway, such as bevacizumab, are standard of care, their long-term efficacy is frequently compromised by drug resistance and compensatory mechanisms [1]. Consequently, characterizing novel regulatory molecules within the tumor microenvironment (TME) is necessary to identify therapeutic targets that enhance current regimens [2]. Aberrant cell surface glycosylation is a hallmark of cancer. Gangliosides, sialic acid-containing glycosphingolipids, are widely expressed on the plasma membrane and modulate cell–cell interactions and signal transduction [3,4,5]. Although some complex gangliosides (e.g., GD2, GD3) are known to promote tumor growth [6,7], GM3 (monosialodihexosylganglioside) plays a unique role [8,9,10]. As the simplest and most abundant ganglioside, GM3 is increasingly recognized as a functional modulator of receptor tyrosine kinases (RTKs) rather than merely a structural component [11,12]. Recent studies suggest that GM3 is a potential therapeutic target for various diseases (includes aortic aneurysms, infectious viruses, and cancer vaccines) [13,14,15]. GM3-based interventions have demonstrated efficacy across diverse pathologies, from cardiovascular and metabolic disorders to malignancies [16].

Here, we review the antitumor potential of GM3, focusing specifically on its anti-angiogenic properties. We outline the biosynthesis and structural characteristics of GM3, examine its crosstalk with key angiogenic pathways (VEGF/VEGFR and EGFR), and discuss its dual role in the TME. Finally, we highlight recent advances in GM3-based therapeutic strategies, including cancer vaccines and synergistic combination therapies, offering a perspective on future clinical applications.

## 2. Ganglioside GM3: Biosynthesis, Structure, and Biological Functions

### 2.1. Biosynthesis and Molecular Characteristics

**Figure 1:**
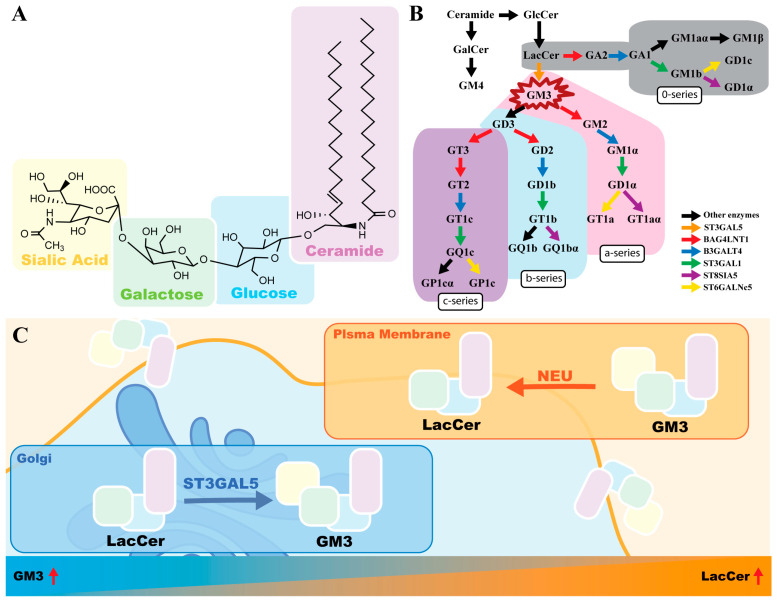
Gangliosides and GM3. (**A**) GM3 structure consists of a ceramide (violet part), a glucose (blue part), a galactose (green part), and a sialic acid (yellow part). (**B**) Biosynthesis and categorization of gangliosides, in which GM3 plays a central role as the simplest ganglioside. Colorized arrows represent different gangliosides synthetase, such as the blue arrows representing B3GALT4 and the black arrow indicating other enzymes. (**C**) The metabolic cycle between GM3 and LacCer. The steady-state level of cell surface GM3 is maintained by the balance between Golgi-resident ST3GAL5 (biosynthesis) and plasma membrane-associated NEU3 (biodegradation).

Gangliosides are sialic acid-containing glycosphingolipids that distribute across vertebrate plasma membranes and mediate cell signaling and membrane organization. Structurally, GM3 comprises a hydrophobic ceramide moiety anchored within the outer leaflet of the lipid bilayer and a hydrophilic oligosaccharide headgroup extending into the extracellular space (Figure 1A). This amphiphilic nature drives GM3 to partition into sphingolipid- and cholesterol-enriched microdomains, or lipid rafts. Within these specialized domains, GM3 regulates transmembrane signaling proteins, most notably RTKs, by modulating their lateral mobility and phosphorylation states [17,18].

As the foundational member of the a-series lineage, GM3 serves as the simplest and most abundant precursor for synthesizing more complex ganglioside species [19,20] (Figure 1B). Its biogenesis primarily occurs in the Golgi apparatus (Figure 1C), where GM3 synthase (ST3GAL5) transfers a sialic acid residue from CMP-sialic acid to lactosylceramide (LacCer) [3]. Rather than being passively determined by continuous biosynthetic cascades, steady-state GM3 levels are actively maintained through spatial coordination with specific degrading enzymes at the cell surface.

### 2.2. Dynamic Regulation of GM3: The LacCer/GM3 Ratio

The physiological abundance of surface GM3 relies heavily on localized degradation mechanisms. Sialidase Neu3, a unique plasma membrane-associated enzyme, specifically targets GM3 to cleave its sialic acid residue, regenerating LacCer directly at the cell surface [21,22]. This enzymatic activity establishes a dynamic LacCer/GM3 ratio that profoundly dictates membrane microdomain organization and subsequent cellular signaling (Figure 1C).

This lipid balance is particularly critical in the tumor microenvironment. GM3 typically functions as a tumor suppressor and anti-angiogenic factor by restricting RTK signaling within lipid rafts. Its aglycone precursor, LacCer, however, actively promotes cell proliferation, migration, and angiogenesis [22]. Aberrant upregulation of Neu3 in various human cancers disrupts this equilibrium, leading to a high LacCer/low GM3 state. By depleting GM3 from membrane microdomains and concurrently enriching them with LacCer, Neu3 dismantles the inhibitory constraints on RTKs, fueling malignant transformation and tumor angiogenesis [23,24]. Therefore, targeting this Neu3-driven shift in the LacCer/GM3 balance thus emerges as a compelling strategy for restraining tumor progression.

### 2.3. Structural Heterogeneity: The Significance of Lipid and Sialic Acid Diversity

**Figure 2:**
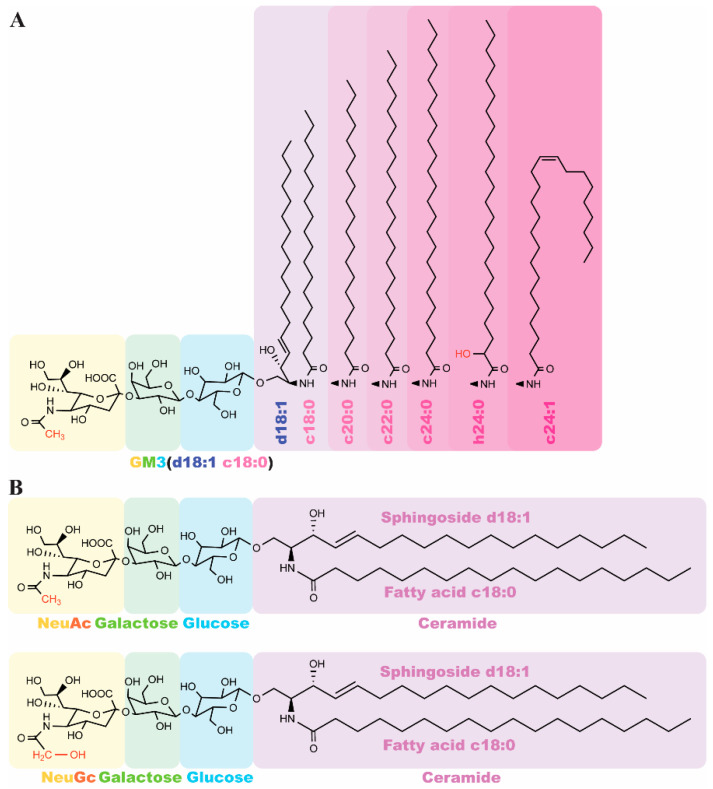
Structures of different gangliosides GM3. (**A**) The molecular diversity of the GM3(d18:1) lipid tail. Variations in the ceramide fatty acid include chain elongation (C18:0 vs. C24:0), α-hydroxylation (h24:0), and desaturation (C24:1). The core structure GM3(d18:1/C18:0) is depicted in purple to illustrate the site of acyl chain substitution. (**B**) Molecular composition of GM3 isoforms. Both variants share a conserved ceramide (violet) and lactose core (glucose: blue; galactose: green). They diverge at the terminal sialic acid (yellow), defined as either N-acetylneuraminic acid (Neu5Ac) or N-glycolylneuraminic acid (Neu5Gc).

The biological functionality of GM3 is affected by its structural heterogeneity, arising from variations in the ceramide moiety and the sialic acid residue. As illustrated in Figure 2A, while the sphingosine base (d18:1) is relatively conserved, the fatty acyl chains exhibit significant diversity in length, saturation, and hydroxylation. The core structure (e.g., d18:1–C18:0, highlighted in purple) can be modified by replacing the fatty acid with longer chains (C24:0), unsaturated variants (C24:1), or α-hydroxylated forms (h24:0).

These specific GM3 isoforms serve as more precise clinical biomarkers than total GM3 levels. For instance, in patients with lymphoma, while total GM3 remains stable, the specific levels of GM3 (d18:1–16:0) and GM3 (d18:1–24:1) are significantly elevated [25]. Similarly, in metabolic research, GM3 (d18:1–h24:1) has been identified as a superior indicator for screening hyperglycemia and dyslipidemia [26]. However, the role of very-long-chain fatty acid (VLCFA) GM3 species, such as h24:0 and h24:1, appears complex, with some studies indicating decreased levels in patients with chronic inflammation and insulin resistance [27,28]. Collectively, these findings suggest that specific GM3 sub-species provide high diagnostic accuracy for various pathological states.

Beyond the lipid tail, the diversity of the sialic acid residue further dictates GM3’s physiological behavior (Figure 2B). The oligosaccharide headgroup, composed of glucose (blue) and galactose (green), is capped by a sialic acid (yellow) that exists primarily as N-acetylneuraminic acid (Neu5Ac) or N-glycolylneuraminic acid (Neu5Gc). While Neu5Ac is the predominant form in healthy humans, Neu5Gc is typically absent due to the evolutionary loss of the CMAH enzyme. While dietary exposure to Neu5Gc is primarily associated with intestinal inflammation and cancer, circulating exogenous Neu5Gc can also be hijacked by distant tumor tissues [29,30]. Hypoxia in TME upregulates specific lysosomal transporters and metabolic pathways, thereby further facilitating the aberrant accumulation of Neu5GcGM3 in non-intestinal solid tumors. Mechanistically, exogenous Neu5Gc from glycoconjugates enters human cells via non-clathrin-mediated pinocytosis/endocytosis and is delivered to lysosomes. There, lysosomal sialidase releases free Neu5Gc, which is subsequently exported into the cytosol by a specific lysosomal sialic acid transporter. Once in the cytosol, it becomes metabolically active and is incorporated into newly synthesized gangliosides [31]. Through this systemic distribution and unique cellular uptake mechanism, non-intestinal malignancies such as breast cancer and melanoma can aberrantly express Neu5GcGM3 on their cell surface, positioning it as a high-priority target for tumor vaccines and targeted immunotherapies.

### 2.4. Physiological Roles and Pathological Implications

Beyond its structural role, GM3 acts as a dynamic rheostat for transmembrane signaling. By partitioning into lipid rafts, GM3 interacts with various growth factor receptors (e.g., EGFR, FGFR, IGFR) and integrins, thereby modulating fundamental cellular processes such as proliferation, differentiation, and adhesion [17,32,33]. Under physiological conditions, GM3 maintains cellular homeostasis by restraining the excessive activation of these RTKs.

Although this review focuses on the tumor microenvironment, briefly examining GM3’s dysregulation in other pathological states—such as metabolic, neurological, and fibrotic disorders—provides essential mechanistic parallels for understanding cancer progression. In metabolic syndromes, elevated plasma GM3 levels are strongly associated with insulin resistance in Type 2 diabetes, primarily through the inhibition of insulin receptor signaling in adipocytes and skeletal muscle [34,35]. Conversely, GM3 deficiency—often resulting from *ST3GAL5* mutations—leads to severe neurodevelopmental pathologies such as epilepsy and salt-and-pepper syndrome, highlighting its importance in neural maturation and synaptic stability [36,37,38]. These insights into GM3’s control over systemic metabolism and neural signaling serve as a valuable reference point for understanding the metabolic reprogramming and neural crosstalk often observed within the tumor microenvironment.

Recent evidence has expanded the therapeutic relevance of GM3 to organ-specific fibrosis and cystic diseases. For instance, GM3 is a critical regulator of the Transforming Growth Factor-β (TGF-β) signaling pathway, which is the primary driver of cardiac fibrosis [34,39,40]. While fibroblasts are essential for wound healing and re-epithelialization following minor injury, severe damage can trigger excessive extracellular matrix (ECM) secretion, leading to fibrotic tissue replacement and organ dysfunction. Notably, GM3 has been shown to inhibit the TGF-β pathway, suggesting its potential as a therapeutic agent to prevent excessive cardiac fibrosis and facilitate chondrocyte regeneration. Importantly, these GM3-mediated mechanisms in tissue fibrosis are highly analogous to the desmoplasia and activation of cancer-associated fibroblasts (CAFs) in the tumor stroma, which are critical drivers of tumor angiogenesis and metastasis. GM3 and its downstream ganglioside derivatives serve as major regulators of cystogenesis; studies indicate that the elimination of GM3 production can have a beneficial effect on slowing cyst formation in polycystic organ diseases [41].

In the specific context of oncology, a vast body of research suggests that increased GM3 content exerts a potent inhibitory effect on various malignancies, including ovarian, colorectal, gastric, and bladder cancers [42,43,44]. These antitumor activities are largely mediated through the dual modulation of the Epidermal Growth Factor (EGF) and Vascular Endothelial Growth Factor (VEGF) signaling pathways, which, respectively, suppress tumor cell proliferation and block pathological angiogenesis. The pleiotropic nature of GM3—ranging from its role in immune cell modulation (such as T cell activation) to its direct interference with oncogenic RTKs—suggests that GM3 supplements and GM3-targeted antibodies have therapeutic potential [45]. Notably, recent advancements have extended this potential into adoptive cell therapy. Sutherland et al. demonstrated a non-genetic approach where T cells and natural killer (NK) cells were chemically conjugated with the 14F7hT antibody targeting Neu5GcGM3. These immune cells exhibited specific tumor-homing capabilities and potent cytotoxicity against Neu5GcGM3-positive cancer cells without the complexities or toxicities often associated with genetic engineering, highlighting a novel therapeutic avenue for GM3-targeted immunotherapy [46].

## 3. GM3-Mediated Suppression of Tumor Malignancy

### 3.1. Suppression of Oncogenic Signaling via RTK Modulation

During malignant transformation, aberrant cell surface glycosylation frequently results in the active shedding of gangliosides into the TME via micelles and membrane vesicles [47]. Unlike complex gangliosides that generally promote progression, GM3 typically functions as a natural inhibitor of tumor growth. As summarized in Table 1, GM3 levels are often inversely correlated with the degree of malignancy, showing higher abundance in benign tumors compared to their invasive counterparts. This tumor-suppressive effect is largely mediated through direct interference with the phosphorylation of multiple RTKs; for instance, in gliomas and bladder cancers, GM3 blocks the activation of EGFR, PDGFR, and FGFR signaling.

Recent studies highlight GM3 as a regulator of proliferation and a potential chemotherapeutic agent for high-grade gliomas [48]. Exogenous GM3 effectively inhibits proliferation and induces apoptosis in primary human brain tumors—including glioblastoma multiforme (GBM), astrocytomas, and oligodendrogliomas—without significantly affecting normal neural cells. In vivo, GM3 administration significantly prolongs symptom-free survival in murine xenograft models and rats with meningeal gliomatosis. Additionally, GM3 rapidly suppresses glioma cell invasion prior to the onset of proliferative arrest or apoptosis. However, ganglioside-mediated effects on invasion remain context-dependent [49]. In certain glioma cell lines, exogenous GM3 paradoxically upregulates the secretion of matrix metalloproteinases (MMP-2 and MMP-9) in vitro, suggesting a capacity to stimulate extracellular matrix degradation under specific conditions. Care must also be taken to distinguish the direct lipid effects of ganglioside GM3 from findings derived from the “GM3” malignant glioma cell line [50], where CIITA-induced growth suppression involves the downregulation of PDGFR-β, the upregulation of p27, and the modulation of the PI3K/Akt and MAPK pathways. At the lipid level, GM3 extensively modulates these same downstream cascades while interfering with HSPA8 phosphorylation to suppress tumor growth.

Similarly, in ovarian cancer, GM3 attenuates invasiveness by upregulating Caveolin-1, which sequesters and inactivates c-Src kinase, leading to the downregulation of malignancy markers like α-SMA. Beyond endogenous signaling, GM3 and its aberrant structural derivatives have emerged as viable immunotherapeutic targets [9]. Immunohistochemical analyses reveal that GM3 is strongly expressed in human ovarian cancer tissues but scarcely in normal tissues. Novel monoclonal antibodies specific to GM3 (such as MAb-1) have demonstrated the ability to significantly suppress cell growth and induce robust antibody-dependent cellular cytotoxicity (ADCC) in ovarian cancer cell lines (e.g., OVHM and ID8), indicating significant translational potential [51]. Tumor-specific variants offer an optimized therapeutic window; for example, NeuGcGM3—a variant incorporating dietary sialic acid due to the human *CMAH* gene mutation—acts as a highly specific neoantigen across various solid tumors. Chimeric antigen receptor (CAR) T cells engineered with single-chain variable fragments (e.g., from the 14F7 antibody) targeting NeuGcGM3 have successfully controlled NeuGcGM3-positive ovarian tumor xenografts in vivo without eliciting off-target toxicity [9]. This NeuGcGM3-targeting strategy is also being integrated into innovative delivery platforms, such as artificial cell-derived vesicles (ACDVs) from cytotoxic T and NK92 cells. Conjugating these vesicles with anti-NeuGcGM3 antibodies (like 14F7hT) significantly enhances tumor cell interaction, internalization, and targeted cytotoxicity. Additionally, other derivatives, such as de-N-acetyl GM3 (d-GM3), are specifically expressed in metastatic tumor cells and are strongly correlated with enhanced metastatic phenotypes through the activation of urokinase-like plasminogen activator (uPA) and MMP-2 [52]. Although primarily characterized in melanoma, the presence of these aberrant GM3 variants underscores the broader significance of targeting ganglioside modifications in highly aggressive and metastatic neoplastic diseases.

The regulatory role of GM3 extends to cell adhesion and metastasis. In human bladder cancer, clinical observations reveal that large amounts of GM3 accumulate predominantly in superficial rather than invasive tumors, suggesting a protective role against tumor penetration. Elevating GM3 levels—whether through the intravesical administration of exogenous GM3 or the genetic overexpression of GM3 synthase—profoundly reduces cell adhesion, motility, invasion, and orthotopic tumor growth while promoting apoptosis. Exogenous GM3 not only reduces EGF-induced phosphorylation to arrest proliferation [44,53] but also acts as a cofactor for CD9 to regulate integrin-mediated motility. Importantly, GM3 intercepts the TGF-β signaling pathway, halting epithelial–mesenchymal transition (EMT). In breast cancer, the perturbation of GM3 levels significantly impacts the PI3K/Akt and NFAT1 signaling axes, crucial drivers of tumor metastasis [54,55,56]. Conversely, in melanoma, specific aberrant derivatives such as de-N-acetyl GM3 (d-GM3) and Neu5GcGM3 act as specific markers for enhanced metastatic phenotypes. These variants can dynamically interact with the uPAR/Integrin α5β1 axis and upregulate urokinase-like plasminogen activator (uPA) to trigger the p38/MAPK cascade and MMP-2 secretion, underscoring the context-dependent nature of ganglioside signaling [52]. Because these abnormal variants and specific molecular conformations (such as the highly immunogenic GM3 lactone) are largely absent in normal tissues, they serve as potent targets for antitumor vaccines and immunotherapy. Targeted interventions, including IgG3 monoclonal antibodies against GM3 lactone (e.g., DH2) and recombinant adeno-associated virus (rAAV)-mediated delivery of anti-Neu5GcGM3 antibodies (e.g., 14F7), have proven highly effective in inducing antibody-dependent cytotoxicity (ADCC) and continuously suppressing melanoma growth in vivo [54,57].

In leukemia models such as K562 cells, GM3 extends beyond growth suppression to actively dictate cell fate [58]. Specifically, GM3 acts as a decisive lineage switch: its accumulation drives the cells exclusively toward a “GM3-rich” megakaryocytoid lineage (marked by platelet peroxidase and GPIIIa expression), whereas its downregulation is associated with erythroid differentiation. This fate-determining capability is highly unique to GM3, as other gangliosides (such as GM1, GM2, or GD1a) fail to induce similar morphological changes [59]. Mechanistically, this accumulation is driven by the PKC/ERK/CREB signaling axis. Activation of ERK leads to CREB binding directly to the GM3 synthase promoter to stimulate transcription—a process strictly independent of the PI3K pathway—further highlighting its role as a lineage-determining factor [58].

From a clinical translation perspective, targeting GM3 as a tumor-associated carbohydrate antigen (TACA) has historically been hindered by its poor immunogenicity [60]. To overcome this, innovative metabolic glycoengineering strategies have been successfully applied in leukemia models. By treating cancer cells with specific biosynthetic precursors (such as ManNPhAc), tumors can be metabolically hijacked to express artificial, highly immunogenic GM3 derivatives (e.g., GM3NPhAc). Subsequent vaccination against these engineered GM3 neo-antigens elicits robust, antigen-specific T cell-dependent cytotoxicity, which significantly suppresses tumor growth and prolongs survival. Additionally, the broader systemic importance of GM3 in clinical therapy is evident in GM3 synthase deficiency models [20]. The profound loss of GM3 structurally alters the cell surface proteome and RTK abundance, paradoxically increasing cellular sensitivity to targeted EGFR inhibitors like erlotinib and revealing a novel therapeutic vulnerability. Beyond its role in signaling modulation and immunotherapy, GM3 holds promise as a diagnostic biomarker. Lipidomic profiling of breast cancer patients has revealed elevated GM3 levels particularly in the luminal B subtype, suggesting that its expression patterns can reflect the specific molecular landscapes of the tumor [61].

**Table 1:** Expression and functional roles of ganglioside GM3 in various malignancies.

Tumor Type	GM3 Expression	Signaling Pathway/Targets	Functional Effects & Mechanisms
Glioma and Glioblastoma [48,49,50,62]	Downregulated; restoration mitigates malignancy	HSPA8; Integrins; EGFR and MAPK p27;	Inhibits invasion and growth via cell–matrix adhesion and HSPA8 phosphorylation; induces p27-mediated cell cycle arrest and modulates PI3K/Akt/MAPK signaling
Ovarian Cancer [9,42,51,63]	Specific antigen (Neu5GcGM3); synthase overexpression reduces invasiveness	Caveolin-1; c-Src; α-SMA; Neu5GcGM3	Suppresses motility via Caveolin-1 upregulation and c-Src inhibition; Neu5GcGM3 targeting (CAR-T, mAbs, ACDVs) elicits antitumor cytotoxicity
Bladder Cancer [44,53]	Inversely correlates with invasiveness; high in superficial tumors vs. lost in invasive types	CD9; TGF-β	Regulates integrin motility as a CD9 cofactor; restoration induces apoptosis and suppresses EMT
Melanoma [52,54,57,64]	High Neu5GcGM3 in metastases; GM3 lactone acts as specific immunogen	uPAR; MMP-2; p38/MAPK; Integrin α5β1; Caveolin-1; FAK/PI3K/Src	Neu5GcGM3 enhances metastasis by activating uPAR/Integrin α5β1 signaling, which triggers the p38/MAPK and MMP-2 secretion. Targeting surface GM3 with hexameric IgM or IgG3 antibodies triggers potent complement/cell-mediated cytotoxicity
Breast Cancer [9,54,55,56]	Elevated GM3/synthase correlates with metastasis; specific antigen (Neu5GcGM3)	PI3K/Akt; NFAT1; 14F7	Drives metastasis via PI3K/Akt and NFAT1 activation; 14F7-CAR T or radiolabeled mAbs enable specific tumor ablation and imaging
Leukemia [20,58,59,60]	Differentiation marker (Megakaryocytic/T cell); upregulated during lineage commitment	PKC/ERK/CREB; GPIIIa; ManNPhAc	Induces differentiation and growth inhibition via PKC/ERK/CREB-mediated synthase expression; metabolic glycoengineering enhances immunotherapeutic sensitivity

### 3.2. Impact on Cell Adhesion and Invasive Phenotype

The transition from a stationary to an invasive phenotype is a hallmark of cancer progression, often facilitated by the loss of inhibitory glycosphingolipids. GM3 regulates this process by stabilizing intercellular adhesion and disrupting the migratory machinery of cancer cells. As highlighted in Table 1, the depletion of GM3 in infiltrating tumors often coincides with the accumulation of longer-chain glycolipids, which favor a more aggressive behavior.

One key mechanism involves the regulation of the urokinase-type plasminogen activator (uPA) and its receptor (uPAR) signaling. GM3 participates in the organization of sphingolipid-rich microdomains (lipid rafts), which are essential for the assembly of uPAR-related invasive complexes. By perturbing these microregions, GM3 effectively dampens the proteolytic activity required for extracellular matrix degradation [11,65].

GM3 importance is also supported by its interaction with scaffold proteins and integrins. In bladder cancer and ovarian carcinoma, GM3 functions as a critical cofactor. Research indicates that GM3 stabilizes a complex involving CD9 and integrin α5β1, which inhibits tumor cell migration. In ovarian cancer specifically, GM3 overexpression has been found to upregulate Caveolin-1, which sequesters c-Src kinase in an inactive state, thereby suppressing cell motility [66].

However, it is crucial to note that structural modifications can reverse these functions. In metastatic melanoma, a specific variant known as Neu5GcGM3 is highly expressed. Unlike native GM3, d-GM3 promotes invasion by activating the p38 MAPK pathway through the uPAR/integrin axis. Conversely, the role of GM3 appears to be tissue-specific, as studies in murine breast cancer models demonstrate that silencing GM3 synthase could inhibit lung metastasis by modulating the PI3K/Akt pathway and NFAT1 expression [67]. Together, these findings suggest that while GM3 generally reinforces cellular cohesion, its effect on invasion depends on the specific signaling context of the tumor.

Furthermore, the structural heterogeneity of GM3 isoforms fundamentally dictates their contradictory roles in cancer progression. A primary source of this heterogeneity lies in the sialic acid headgroup. Humans are genetically deficient in synthesizing Neu5Gc due to an irreversible mutation in the *CMAH* gene [68]. Consequently, the incorporation of dietary-derived Neu5Gc introduces a seemingly minor structural variation—a single additional oxygen atom at the C-5 position—that drastically alters GM3 function [30]. While endogenous Neu5AcGM3 typically exerts tumor-suppressive effects by maintaining tissue homeostasis and dampening RTK signaling, Neu5GcGM3 acts as a potent pro-tumorigenic driver. The presence of Neu5GcGM3, particularly when interacting with circulating anti-Neu5Gc antibodies, triggers chronic inflammation (xenosialitis) that actively promotes tumor progression and immune evasion [69].

Beyond sialic acid modification, the heterogeneity of the ceramide lipid tail—such as variations in acyl chain length (e.g., C16:0 vs. C24:0) and degree of hydroxylation—further contributes to these contradictory functions. Distinct ceramide structures dictate the specific partitioning of GM3 isoforms within diverse lipid raft microdomains, thereby selectively recruiting or repelling different signalosome complexes [70]. Thus, the net biological effect of GM3 in the tumor microenvironment is not monolithic, but rather a dynamic outcome governed by the balance of these structurally distinct, and often functionally opposing, isoforms.

## 4. GM3 as a Potent Inhibitor of Tumor Angiogenesis

Angiogenesis, the formation of new blood vessels from pre-existing ones, is an early hallmark of malignant transformation and a prerequisite for tumor expansion beyond a few millimeters. This complex process is driven by a balance between pro-angiogenic and anti-angiogenic factors within the TME. While complex gangliosides like GM2 and GD1a are known to promote a pro-angiogenic environment, GM3 exerts a distinctive inhibitory influence on vascular remodeling.

### 4.1. Interference with the VEGF/HIF-1α Signaling Axis

Angiogenesis is a complex and dynamic process essential for tumor growth and metastasis, regulated by a delicate balance of biomolecules including growth factors and chemokines. While the VEGF pathway is established as the primary driver of tumor-associated neovascularization—prompting the widespread clinical use of monoclonal antibodies and tyrosine kinase inhibitors (TKIs)—the efficacy of these therapies is often limited by acquired drug resistance and tumor recurrence [71]. GM3 functions as a physiological disruptor of the angiogenic axis. Unlike standard agents that primarily block receptor activity, GM3 targets both the production of angiogenic ligands and their upstream transcriptional regulators. A central mechanism underlying this inhibition is the modulation of hypoxia-inducible factor 1-alpha (HIF-1α). Under the hypoxic conditions typical of the tumor microenvironment, GM3 prevents the stabilization of HIF-1α, thereby abrogating the transcriptional regulation of VEGF and other pro-angiogenic genes.

GM3 weakens these metabolic events by blocking the nuclear translocation of HIF-1α. A reciprocal relationship exists where HIF-1α can suppress ST3GAL5 expression, suggesting that GM3 loss may facilitate tumor progression to sustain an angiogenic switch [72]. Crucially, reversing this loss can halt tumor progression, as evidenced by studies using the GM3 synthase up-regulator, valproic acid (VPA). In A431 epidermoid carcinoma and U87MG glioma cells, VPA treatment induced an approximate 8-fold increase in ST3GAL5 expression, resulting in significant GM3 accumulation [73]. This restoration of GM3 was directly linked to the inhibition of EGFR phosphorylation and cell proliferation—a conclusion further validated when the effects were reversed by blocking glucosylceramide synthesis. Thus, by restoring GM3, therapeutic agents can target a broad signaling network, leading to the concurrent reduction in VEGF-mediated angiogenesis and EGFR-dependent growth.

### 4.2. Direct Blockade of the VEGFR2 Receptor

**Figure 3:**
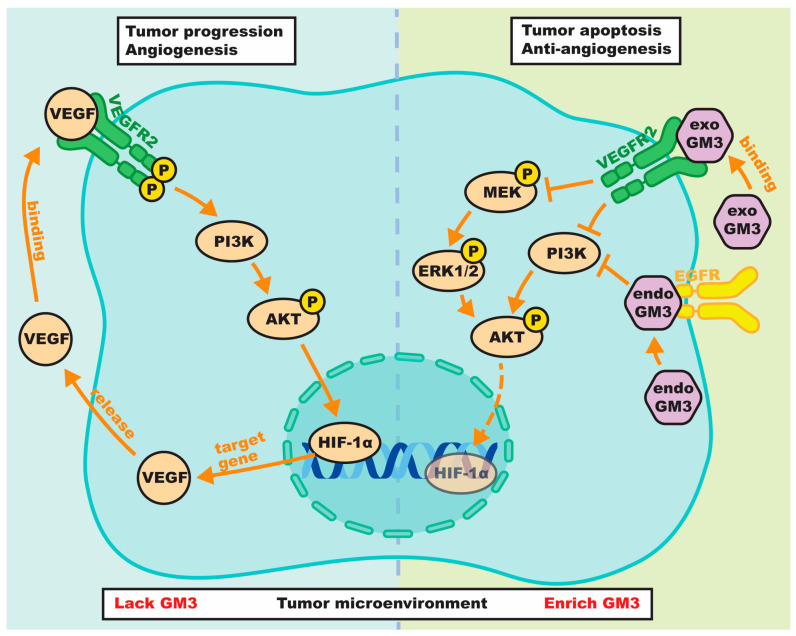
The effect of GM3 on the VEGF signaling pathway. Upon VEGF binding, VEGFR-2 undergoes dimerization and tyrosine phosphorylation, triggering downstream signaling cascades including MEK/ERK1/2, PI3K/Akt, and HIF-1α pathways that promote cell survival, migration, and vascular permeability. Exogenous GM3 inhibits these processes by interacting with the immunoglobulin-like domains 2 and 3 of VEGFR-2 via its sialic acid moiety. Additionally, endogenous GM3 interferes with VEGFR-2 dimerization, leading to suppressed HIF-1α nuclear transcription. Both mechanisms synergistically inhibit tumor-associated angiogenesis.

Direct interference with the vascular endothelial growth factor receptor 2 (VEGFR2) represents another mechanism of regulation by GM3. As illustrated in Figure 3, the activation of VEGFR2 typically requires ligand binding followed by receptor dimerization and subsequent tyrosine phosphorylation. This cascade activates multiple downstream pathways, including MEK-ERK1/2 and PI3K-Akt, which collectively promote cell survival, migration, and increased vascular permeability.

The inhibitory action of GM3 on this axis is twofold, involving both exogenous and endogenous mechanisms that synergistically suppress angiogenic signaling. Exogenous GM3 within the tumor microenvironment directly interacts with the extracellular immunoglobulin-like structural domains of VEGFR2, specifically binding to amino acids in domains 2 and 3 [74]. This interaction pre-empts the VEGF ligand-binding site, effectively preventing receptor dimerization and reducing the interaction between VEGFR2 and its ligand. Notably, this blockade counteracts pro-angiogenic signaling, as GM3 has been shown to neutralize the proliferative effects of complex gangliosides like GD1a.

Concurrently, endogenous GM3 located within the plasma membrane creates an internal blockade that prevents the relay of activating signals to cytoplasmic mediators. This regulation is highly specific: the depletion of GM3 using synthesis inhibitors (e.g., NB-DNJ) results in the hyper-phosphorylation of VEGFR2 and Akt, a phenotype that is fully reversible upon GM3 replenishment [75]. Functionally, these dual mechanisms translate into marked suppression of endothelial cell migration and microvessel permeability. These findings demonstrate that GM3 administration blocks neovascularization and inhibits primary tumor growth.

As a result of these dual approaches, the phosphorylation of ERK and Akt is significantly inhibited. This suppression of the kinase network is not limited to the VEGF axis but reflects a broader regulatory role of GM3 on the cellular kinase. Mechanistic studies, particularly in keratinocyte models, revealed that GM3 also negatively regulated the insulin receptor (IR) and insulin-like growth factor-1 receptor (IGF-1R) pathways [76]. Specifically, GM3 acts by hindering the formation of the signaling complex involving IR, IGF-1R, and insulin receptor substrate 1 (IRS-1), thereby blocking the downstream activation of PI3K/Akt.

The consequence of this broad kinase inhibition is a decisive shift in cell fate. Disruption of these survival signals (ERK1/2, PI3K, Akt) by GM3 or ganglioside-targeting agents has been shown to trigger pro-apoptotic cascades, characterized by the upregulation of caspase-3 and BAX [77]. Consequently, the restoration of GM3 levels induces metabolic suppression and apoptosis in tumor cells. Importantly, this inhibitory efficacy is not confined to the tumor cells themselves but extends to the broader angiogenic microenvironment.

### 4.3. Broader Impact on Angiogenic Microenvironment

Beyond direct receptor interference, GM3 reshapes the vascular niche by modulating auxiliary signaling molecules. GM3 has been found to dose-dependently reduce NO levels in macrophages; since NO production is known to facilitate vasodilation and attract existing capillaries, its reduction further contributes to the localized anti-vascular effect [78]. In vivo models further demonstrate that environments enriched with GM3 result in sparsely vascularized and extensively necrotic solid tumors, contrasting sharply with the dense vascular networks observed in GM3-deficient tissues [79].

In addition to its antitumor role, GM3 exhibits a fundamental protective function in vascular homeostasis. Our group previously demonstrated that exogenous GM3 effectively inhibited atherosclerosis through a multi-step mechanism. Specifically, instead of merely acting as a passive lipid, GM3 incorporation altered the physicochemical properties of low-density lipoprotein (LDL). This modification reduces LDL susceptibility to oxidation and macrophage uptake, while simultaneously impairing endothelial monocyte adhesion [80]. Extending this vascular protective profile, recent findings by Zhang et al. revealed that GM3 also prevented abdominal aortic aneurysm (AAA) progression [13]. This was achieved by blocking transferrin receptor 1 (TFR1)-mediated iron uptake, thereby suppressing ferroptosis in vascular smooth muscle cells. Collectively, these findings indicate that GM3 maintains vascular homeostasis by regulating lipid metabolism, inflammation, and cell survival.

## 5. Therapeutic Strategies Targeting GM3

Given the tumor-suppressive function of GM3, several immunotherapeutic strategies, including cancer vaccines and monoclonal antibodies, have been developed. Recent advances highlight that gangliosides act as functional components of membrane lipid rafts, making these microdomains essential therapeutic targets for disrupting cancer cell communication [81]. Natural GM3 is weakly immunogenic and typically elicits only IgM responses. However, chemically modified GM3 derivatives demonstrate improved immunogenicity. For example, N-phenylacetylated derivatives (GM3NPhAc) induce high titers of both IgG and IgM antibodies with high affinity and minimal cross-reactivity to endogenous GM3 [60]. Hexameric IgM antibodies have been engineered to specifically target GM3-positive melanoma cells [82,83,84].

**Figure 4:**
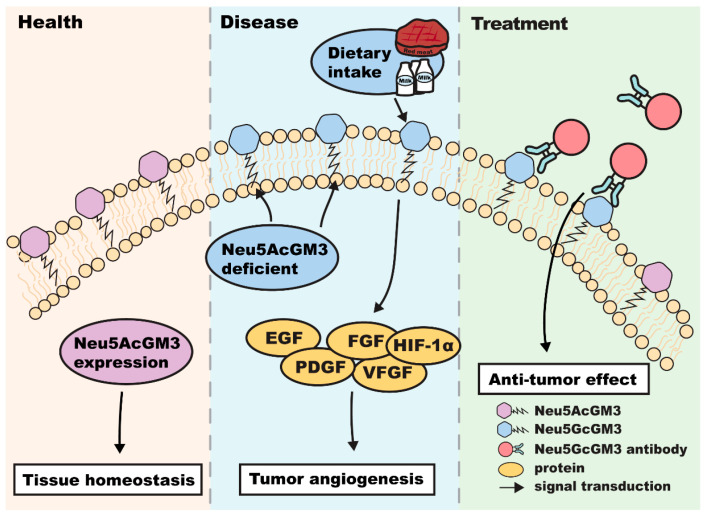
The mechanism of Neu5GcGM3 accumulation and antibody-mediated inhibition. In malignant states, the upregulation of Neu5GcGM3 on the cell surface is driven by the metabolic incorporation of exogenous N-glycolylneuraminic acid (derived from dietary sources such as red meat and dairy) and the simultaneous downregulation of enzymes responsible for Neu5AcGM3 synthesis. This modification enhances receptor tyrosine kinase (RTK) signaling (e.g., EGFR, PDGFR, FGFR). Anti-Neu5GcGM3 antibodies (e.g., 14F7) selectively bind to these neo-antigens, blocking downstream oncogenic signaling and triggering immune responses.

Another focus on this field is targeting N-glycolyl GM3 (Neu5GcGM3), a non-human sialic acid variant that acts as a neo-antigen [85]. As illustrated in Figure 4, the pathological accumulation of Neu5GcGM3 on the cell surface is driven by the metabolic incorporation of exogenous N-glycolylneuraminic acid—derived from dietary sources such as red meat and dairy—combined with a deficiency in the enzymes responsible for Neu5AcGM3 synthesis [86]. This altered sialylation profile facilitates the hyperphosphorylation of RTKs, including EGFR, PDGFR, and FGFR, which ultimately accelerates malignant transformation and tumor development [30]. To inhibit this signaling, humanized monoclonal antibodies, such as 14F7, have been developed to selectively bind to Neu5GcGM3. These antibodies block RTK signaling pathways and induce immune-mediated cytotoxicity against cancer cells [29,87,88].

The Neu5GcGM3/very-small-size proteoliposome (VSSP) vaccine has shown efficacy in clinical trials [89]. In advanced melanoma patients, the VSSP vaccine extended survival to over 20 months [90]. Similar strategies using Neu5GcGM3 complexed with *Neisseria meningitidis* outer membrane proteins have been tested in breast cancer [91]. Glyco-engineered mouse models (*β3Gn-T5* knockout) have confirmed the feasibility of generating high-affinity IgG antibodies against Neu5GcGM3 [51].

Beyond its standalone effects, GM3 can function as a targeting or stabilizing moiety in complex drug delivery systems. Our study demonstrated that GM3-functionalized reconstituted high-density lipoproteins (rHDLs) significantly enhanced the anti-atherosclerotic efficacy of statins through improved lesion targeting [92]. It suggests that GM3-based nanoplatforms could similarly be employed in oncology to co-deliver anti-angiogenic and chemotherapeutic agents, achieving synergistic tumor suppression.

However, anti-ganglioside therapy carries potential risks. The induction of anti-ganglioside antibodies (AGAs) has been linked to neurological conditions such as Guillain–Barré syndrome, an autoimmune polyneuropathy [93]. Since certain microbial infections can mimic ganglioside structures and trigger cross-reactive autoantibodies [94], distinguishing between therapeutic and pathological immune responses is critical for the safety of GM3-targeted therapies.

## 6. Conclusions and Future Perspectives

It is important to highlight the unique scope of this review. While recent comprehensive reviews have predominantly focused on the clinical landscape of Neu5Gc-containing glycoconjugates and their applications as tumor-associated antigens [30], our review offers a fundamentally distinct perspective. The core novelty of this work lies in conceptually repositioning GM3 and its structural isoforms not merely as passive structural biomarkers, but as dynamic regulatory hubs that specifically dictate tumor angiogenesis and profoundly remodel the TME. Ganglioside GM3 functions well beyond simple cell–cell recognition; by acting as a natural antagonist to RTKs—including EGFR, PDGFR, and VEGFR2—it actively inhibits the oncogenic signaling that drives tumor proliferation and invasion. Specifically, GM3 disrupts the VEGF/HIF-1α axis at both transcriptional and receptor levels, thereby suppressing tumor angiogenesis and vascular expansion.

Despite these established mechanisms, several highly controversial issues remain in the field. The most prominent debate centers on the paradoxical, context-dependent functions of GM3. Distinguishing between the universally tumor-suppressive native Neu5AcGM3 and the aberrantly expressed Neu5GcGM3 variant is essential. While endogenous Neu5AcGM3 dampens oncogenic signaling, the incorporation of non-human Neu5Gc, or specific variations in the ceramide lipid tail, can completely reverse these effects, driving immune evasion and tumor metastasis. Whether these contradictory roles are solely dictated by the structural heterogeneity of these GM3 isoforms, or if they are largely shaped by their specific cellular origins within the TME (e.g., cancer cells vs. cancer-associated fibroblasts), remains a subject of intense debate.

Translating these biological insights into clinical therapeutics highlights critical gaps of knowledge and translational hurdles, particularly regarding inadequate drug targeting and off-target toxicity. Meanwhile, novel nanomedicine strategies, such as bioinspired membrane vesicles, are continuously emerging to overcome these generic delivery challenges [95,96]. The identification of Neu5GcGM3 as a tumor-specific neo-antigen has provided a promising target for immunotherapy. Strategies utilizing humanized antibodies (e.g., 14F7) and vaccine formulations (e.g., VSSP) demonstrate the feasibility of targeting Neu5GcGM3-expressing malignancies. This approach potentially offers a dual mechanism: activating the immune system while simultaneously inhibiting oncogenic signaling.

However, clinical application faces challenges. Distinguishing between the tumor-suppressive Neu5AcGM3 and the targetable Neu5GcGM3 variant is essential to avoid off-target effects. Future research should prioritize the development of advanced delivery systems, such as lipid-based nanoparticles or liposomes, to enhance the bioavailability and stability of exogenous GM3 analogs. To overcome the biological barriers of GM3 delivery, biomimetic nanocarriers like rHDL have been developed to provide enhanced stability and lesion specificity. These systems can be engineered for pH-responsive release, capitalizing on the acidic microenvironment to trigger localized drug liberation [97]. Given the characteristic acidosis of the TME, such pH-sensitive delivery strategies represent a promising avenue for maximizing the local anti-angiogenic impact of GM3 while minimizing systemic side effects. Notably, rigorous monitoring for autoimmune risks, particularly anti-ganglioside antibody-induced neuropathies (e.g., Guillain–Barré syndrome) is required. Combining GM3-targeted immunotherapies with conventional anti-angiogenic agents may offer a synergistic strategy to inhibit tumor growth and metastasis effectively.

## Data Availability

No new data were created or analyzed in this study.

## Abbreviations

The following abbreviations are used in this manuscript:

α-SMA	Alpha-smooth muscle actin
CAFs	Cancer-associated fibroblasts
CMP	Cytidine monophosphate
ECM	Extracellular matrix
EGF	Epidermal Growth Factor
EMT	Epithelial–mesenchymal transition
GM3	Monosialodihexosylganglioside
HIF-1α	Hypoxia-inducible factor 1-alpha
IGFR	Insulin-like growth factor receptor
LacCer	Lactosylceramide
Neu3	Sialidase Neu3
Neu5Ac	N-acetylneuraminic acid
Neu5Gc	N-glycolylneuraminic acid
NK	Natural killer
PDGFR	Platelet-Derived Growth Factor Receptor
RTKs	Receptor tyrosine kinases
ST3GAL5	GM3 synthase
TGF-β	Transforming growth factor-β
TME	Tumor microenvironment
uPAR	Urokinase-type plasminogen activator receptor
VEGF	Vascular Endothelial Growth Factor
VEGFR/VEGFR2	Vascular Endothelial Growth Factor Receptor (2)
VSSP	Very-small-size proteoliposome
